# Association of Ischemic Core Imaging Biomarkers With Post-Thrombectomy Clinical Outcomes in the MR CLEAN Registry

**DOI:** 10.3389/fneur.2021.771367

**Published:** 2022-01-10

**Authors:** Miou S. Koopman, Jan W. Hoving, Manon Kappelhof, Olvert A. Berkhemer, Ludo F. M. Beenen, Wim H. van Zwam, Hugo W. A. M. de Jong, Jan Willem Dankbaar, Diederik W. J. Dippel, Jonathan M. Coutinho, Henk A. Marquering, Bart J. Emmer, Charles B. L. M. Majoie, for the MR CLEAN Registry Investigators, Diederik W. J. Dippel

**Affiliations:** ^1^Department of Radiology and Nuclear Medicine, Amsterdam University Medical Centers, University of Amsterdam, Amsterdam, Netherlands; ^2^Department of Radiology and Nuclear Medicine, Erasmus University Medical Center, Rotterdam, Netherlands; ^3^Department of Radiology, Cardiovascular Research Institute Maastricht (CARIM), Maastricht University Medical Center+, Maastricht, Netherlands; ^4^Department of Radiology, University Medical Center Utrecht, Utrecht, Netherlands; ^5^Department of Neurology, Erasmus University Medical Center, Rotterdam, Netherlands; ^6^Department of Neurology, Amsterdam University Medical Centers, University of Amsterdam, Amsterdam, Netherlands; ^7^Department of Biomedical Engineering and Physics, Amsterdam University Medical Centers, University of Amsterdam, Amsterdam, Netherlands

**Keywords:** CT perfusion (CTP), ischemic core, thrombectomy, stroke, alberta stroke program early CT score (ASPECTS), collaterals

## Abstract

**Background:** A considerable proportion of acute ischemic stroke patients treated with endovascular thrombectomy (EVT) are dead or severely disabled at 3 months despite successful reperfusion. Ischemic core imaging biomarkers may help to identify patients who are more likely to have a poor outcome after endovascular thrombectomy (EVT) despite successful reperfusion. We studied the association of CT perfusion-(CTP), CT angiography-(CTA), and non-contrast CT-(NCCT) based imaging markers with poor outcome in patients who underwent EVT in daily clinical practice.

**Methods:** We included EVT-treated patients (July 2016–November 2017) with an anterior circulation occlusion from the Multicenter Randomized Clinical Trial of Endovascular Treatment for Acute Ischemic Stroke in the Netherlands (MR CLEAN) Registry with available baseline CTP, CTA, and NCCT. We used multivariable binary and ordinal logistic regression to analyze the association of CTP ischemic core volume, CTA-Collateral Score (CTA-CS), and Alberta Stroke Program Early CT Score (ASPECTS) with poor outcome (modified Rankin Scale score (mRS) 5-6) and likelihood of having a lower score on the mRS at 90 days.

**Results:** In 201 patients, median core volume was 13 (IQR 5-41) mL. Median ASPECTS was 9 (IQR 8-10). Most patients had grade 2 (83/201; 42%) or grade 3 (28/201; 14%) collaterals. CTP ischemic core volume was associated with poor outcome [aOR per 10 mL 1.02 (95%CI 1.01–1.04)] and lower likelihood of having a lower score on the mRS at 90 days [aOR per 10 mL 0.85 (95% CI 0.78–0.93)]. In multivariable analysis, neither CTA-CS nor ASPECTS were significantly associated with poor outcome or the likelihood of having a lower mRS.

**Conclusion:** In our population of patients treated with EVT in daily clinical practice, CTP ischemic core volume is associated with poor outcome and lower likelihood of shift toward better outcome in contrast to either CTA-CS or ASPECTS.

## Introduction

The efficacy of endovascular thrombectomy (EVT) is well-established for eligible patients with acute ischemic stroke due to anterior circulation large vessel occlusion (LVO) in both the early (0–6 h) and late (6–24 h) time window (1–7). Most of the early time window trials used non-contrast computed tomography (NCCT) and computed tomography angiography (CTA) to select patients, whereas the late time window trials used computed tomography perfusion (CTP) or magnetic resonance imaging (MRI) to select patients based on ischemic core volumes. In daily clinical practice, the most commonly used imaging parameters to evaluate patients for EVT are the Alberta Stroke Program Early CT Score (ASPECTS) on NCCT, occlusion location and collateral score on CTA (CTA-CS), and the estimated ischemic core volume on CTP. A pooled meta-analysis of the Highly Effective Reperfusion Evaluated in Multiple Endovascular Stroke (HERMES) collaboration data showed an independent association of CTP ischemic core volume and patients' functional outcome, although modification of benefit from EVT over standard care was not shown (8). The AHA/ASA and ESO/ESMINT guidelines currently do not recommend CTP for patient selection for EVT in the early time window (9, 10).

In the late time window, the DAWN and DEFUSE-3 trials showed benefit from EVT in patients who were selected using CTP or MRI (6, 7). Therefore, the AHA/ASA and ESO/ESMINT guidelines do recommend the use of advanced imaging for selection of EVT in the late time window (9, 10). The above-mentioned guidelines are based on data from trials with predetermined NCCT, CTA, or CTP in- and exclusion criteria.

The treatment effect of EVT is robust and persists in patients with premorbid disability (11). However, less than half of the acute ischemic stroke patients fail to recover to living independently at 3 months after EVT in both the early and late time window (12). If imaging biomarkers could identify patients with a high mortality risk or identify patients who are likely to be severely disabled despite successful EVT, this could potentially support physicians in their decision to treat or not to treat a patient. Consequently, this would enable an individualized, patient- and physiology-based stroke treatment selection rather than selection based on time only (13, 14).

Hence, in this *post-hoc* analysis of the Multicenter Randomized Controlled Trial of Endovascular Treatment for Acute Ischemic Stroke in the Netherlands (MR CLEAN) Registry, we aim to assess the association of CTP-, CTA-, and NCCT-based imaging biomarkers with poor functional outcome in EVT-treated patients with acute ischemic stroke.

## Materials and Methods

### Patient Selection

**Figure 1:**
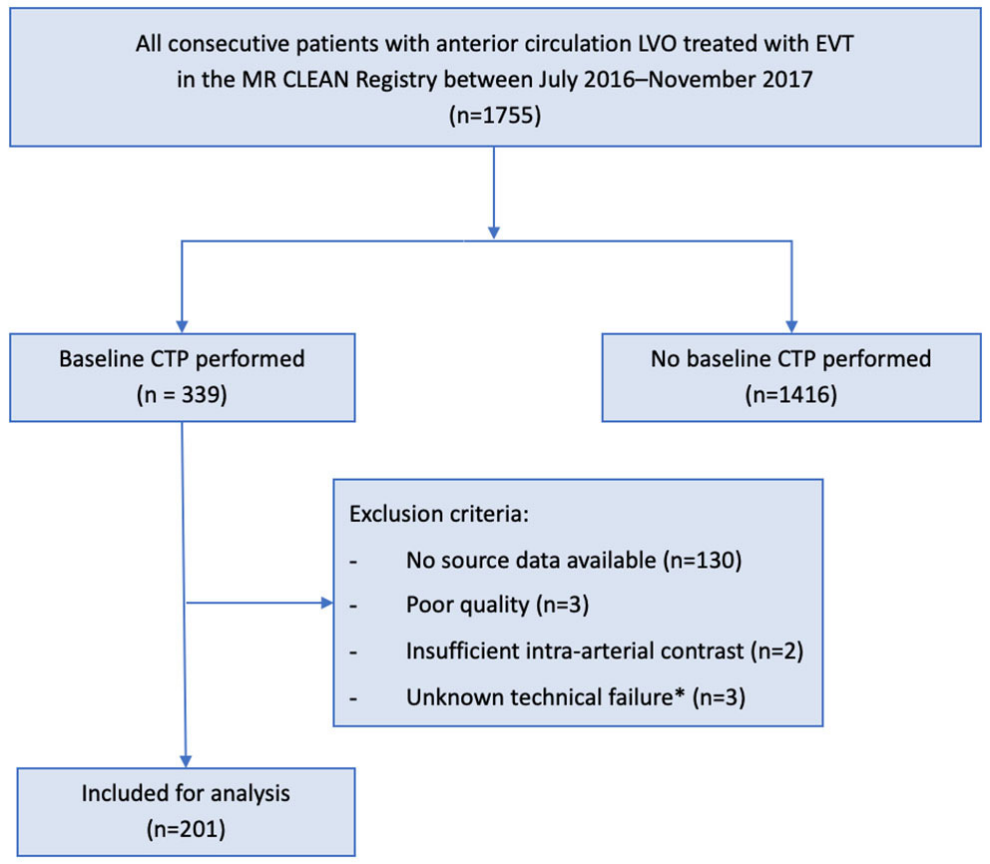
Flowchart for patient selection. EVT, endovascular treatment; CTP, computed tomography-perfusion; MR CLEAN, Multicenter Randomized Controlled Trial of Endovascular Treatment for Acute Ischemic Stroke in the Netherlands. *Unknown scan data issue causing failure in post-processing software functioning.

We used individual clinical and imaging data from patients in the MR CLEAN Registry (July 2016–November 2017) with available baseline CTP data in the 0–24-h time window. The MR CLEAN Registry was an observational, prospective registry of all patients undergoing EVT for acute ischemic stroke in the Netherlands (12). We included patients with a proximal occlusion of the anterior circulation aged >18 years old and treated in a MR CLEAN trial center. Patients were excluded if CTP quality was poor due to movement artifacts, insufficient intra-arterial contrast, or technical failure during post-processing. A schematic representation of our patient selection is given in Figure 1.

### Baseline Imaging Assessment

NCCT and CTA data were assessed by an independent core laboratory of (neuro) radiologists (12). Observers were blinded to all clinical information except occlusion side. Observers evaluated occlusion location and CTA-CS on CTA, ASPECTS on NCCT, and expanded treatment in cerebral ischemia (eTICI) score on post-treatment digital subtraction angiography (DSA). CTA-CS ranges from 0 (absent collaterals) to 3 (excellent collaterals). Poor CTA-CS was defined as CTA-CS 0-1. ASPECTS is a 10-point quantitative NCCT score to quantify early ischemic changes on NCCT. Poor ASPECTS was defined as ASPECTS 0-5. The eTICI score ranges from 0 (no antegrade reperfusion) to 3 (complete antegrade reperfusion).

### CTP Acquisition, Post-processing, and Visual Quality Assessment

CTP images were acquired according to local protocols per site. CTP data were centrally post-processed using syngo.via CT Neuro Perfusion (version VB30A, Siemens Healthineers, Forchheim, Germany). The ischemic core was estimated as CBV <1.2 mL/100 mL and critically hypoperfused tissue volume (including penumbra) as CBF <27 mL/100 mL/min. A default smoothing filter was applied (15). Visual quality assessment of the post-processed data was performed by an experienced neuroradiologist (CBLMM; >15 years of experience). Craniocaudal cropping of the ischemic core (available by default in syngo.via) was performed by two readers (MSK and JWH) to remove obvious artifacts at the skull base level. Penumbra was defined as critically hypoperfused volume minus ischemic core volume. The mismatch ratio was calculated as the critically hypoperfused volume divided by the ischemic core volume. Presence of a target mismatch profile (TMM) was defined using the EXTEND-IA criteria: mismatch ratio >1.2, penumbra volume >10 mL, and ischemic core volume <70 mL (2).

### Outcomes

The primary outcome was poor outcome, defined as a 90-day modified Rankin Scale (mRS) score 5–6. Secondary outcomes were likelihood of having a lower mRS at 90 days, functional independence (mRS 0-2), and reperfusion after EVT (eTICI). Safety outcomes were mortality and symptomatic intracranial hemorrhage (sICH) – defined as an increase of ≥4 points on the National Institutes of Health Stroke Scale (NIHSS) and confirmation of intracranial hemorrhage on neuroimaging.

### Missing Data

We used multiple imputation with five imputations to handle missing data. We adjusted our analyses for the following variables: age, baseline NIHSS score, pre-stroke mRS, baseline ASPECTS, occlusion segment, collateral status, time from symptom onset to groin, eTICI, and 90-day mRS score. The presented baseline, imaging, and outcome characteristics are crude and not imputed. We reported the number of known values for each baseline and outcome variable.

### Statistical Analysis

Baseline, imaging, and outcome characteristics were reported using summary statistics and non-parametric tests appropriate to the type of data and were compared to the overall MR CLEAN Registry population. We used univariable and multivariable logistic regression to analyze the associations with poor outcome and functional independence of the CTP ischemic core volume, penumbra volume, CTA-CS, and ASPECTS for each biomarker separately. We used ordinal logistic regression for the likelihood of having a lower mRS at 90 days. We adjusted for the following pre-defined variables: age, sex, baseline National Institutes of Health Stroke Scale (NIHSS) score, prestroke mRS, administration of intravenous alteplase, time from stroke onset to groin, and site of vessel occlusion.

We investigated if the association of successful reperfusion (defined as eTICI 2b-3) with a shift toward better outcome was modified by CTP ischemic core volume, CTA-CS, and ASPECTS by adding interaction terms (CTP ischemic core volume^*^successful reperfusion; CTA-CS^*^successful reperfusion; ASPECTS^*^successful reperfusion) to the multivariable ordinal regression models. We adjusted for the above-mentioned variables.

We performed an exploratory subgroup analysis to determine the association of dichotomized CTP ischemic core volume (≥70 and ≥50 vs. <70 and <50 mL) with the primary and secondary outcomes. Finally, we assessed whether the absence of a TMM was associated with poor outcome.

We reported (adjusted) odds ratios [(a)ORs] with 95% confidence intervals (95% CI) and considered a two-sided *p* < 0.05 statistically significant. Statistical analyses were performed using R (R Statistical Software, V3.5.0, R: A language and environment for statistical computing, R Foundation for Statistical Computing, Vienna, Austria).

### Protocol Approval and Patient Consent

The central medical ethics committee of the Erasmus MC, Rotterdam, the Netherlands, evaluated the study protocol of the MR CLEAN Registry and granted permission to carry out the study as a registry (MEC-2014-235).

## Results

We included 201 patients for analysis. A detailed overview of the baseline characteristics and outcomes is given in Table 1. Median age was 71 years. Most patients were female (58%). Median onset-to-imaging time was 79 min. Of the patients, seven patients (3%) received baseline imaging beyond 6 h after onset (median 440 min). Twelve patients (6%) had an onset-to-groin time >6 h (median 485 min). At 90 days, 51 (25%) patients had a poor functional outcome (mRS 5–6). The distribution of occlusion location, CTA-CS, and ASPECTS was comparable to the overall MR CLEAN Registry cohort (Table 1). The distributions of CTP ischemic core volumes per ASPECTS and CTA-CS category are given in the Supplementary Figures I, II.

**Table 1:** Baseline characteristics and outcome of the MR CLEAN Registry CTP subgroup compared to the overall MR CLEAN Registry cohort.

	**MR CLEAN Registry CTP subgroup (*N* = 201)**	**Overall MR CLEAN Registry (*N* = 1,755)**	***P-*value**
**Clinical**			
Age (yr) – median (IQR)	71 (56–80)	72 (62-81)	0.1
Female – *n* (%)	118 (59)	889 (51)	0.03
NIHSS score – median (IQR)	16 (12–20) [*N* = 198]	16 (11–19)	0.1
[known in]			
Transfer from primary stroke center – *n* (%)	20 (10)	962 (55)	<0.01
IVT administered – *n* (%)	144 (72)	1,282 (73)	0.7
Onset-to-imaging time (min) – median (IQR) [known in]	79 (79–137) [*N* = 161]	76(53–128) [*N* = 1,279]	0.8
Onset-to-groin time (min) – median (IQR) [known in]	153 (120–222) [*N* = 197]	185 (144–243) [*N* = 1,740]	<0.01
**Imaging**			
Occlusion location on baseline CTA – *n* (%) [known in]	[*N* = 199]	[*N* = 1,657]	0.3
*Intracranial ICA*	6 (3)	76 (5)	
*ICA-T*	35 (18)	342 (20)	
*M1*	121 (61)	974 (58)	
*M2*	36 (18)	295 (17)	
*Other*	1 (1)	6 (0.3)	
ASPECTS – median (IQR) [known in]	9(8–10) [*N* = 193]	9 (8–10) [*N* = 1,713]	0.8
CTA-CS – *n* (%) [known in]	[*N* = 198]	[*N* = 1,693]	0.07
*0*	8 (4)	89 (5)	
*1*	79 (40)	635 (38)	
*2*	83 (42)	643 (39)	
*3*	28 (14)	290 (18)	
Baseline ischemic core volume on CTP (mL) – median (IQR)	13 (5–40)	NA	
Baseline penumbra volume on CTP (mL) – median (IQR)	96 (56–123)	NA	
eTICI – *n* (%) [known in]	[*N* = 193]	[*N* = 1,688]	0.5
*0*	23 (12)	263 (16)	
*1*	4 (2)	45 (3)	
*2a*	29 (15)	276 (16)	
*2b*	41 (21)	399 (24)	
*2c*	28 (15)	214 (13)	
*3*	68 (35)	491 (29)	
**Outcomes**			
Poor functional outcome (mRS 5-6)– *n* (%)	51 (25)	574 (33)	0.7
Functional independence (mRS 0-2)– *n* (%)	90 (45)	708 (40)	0.2
Mortality at 90 days – *n* (%)	38 (19)	479 (27)	0.02
sICH – *n* (%)	8 (4)	104 (6)	0.3

*ASPECTS, Alberta Stroke Program Early CT Score; CTA-CS, Computed Tomography Angiography Collateral Score; CTP, Computed Tomography Perfusion; ICA, internal carotid artery; ICA-T, internal carotid artery terminus; IVT, IV alteplase; IQR, interquartile range; mRS, modified Rankin Score; NIHSS, National Institute of Health Stroke Scale; sICH, symptomatic intracranial hemorrhage*.

*If the [known in] number is not shown, the variable was known in all patients*.

### Association of CTP Ischemic Core Volume With Outcome

**Figure 2:**
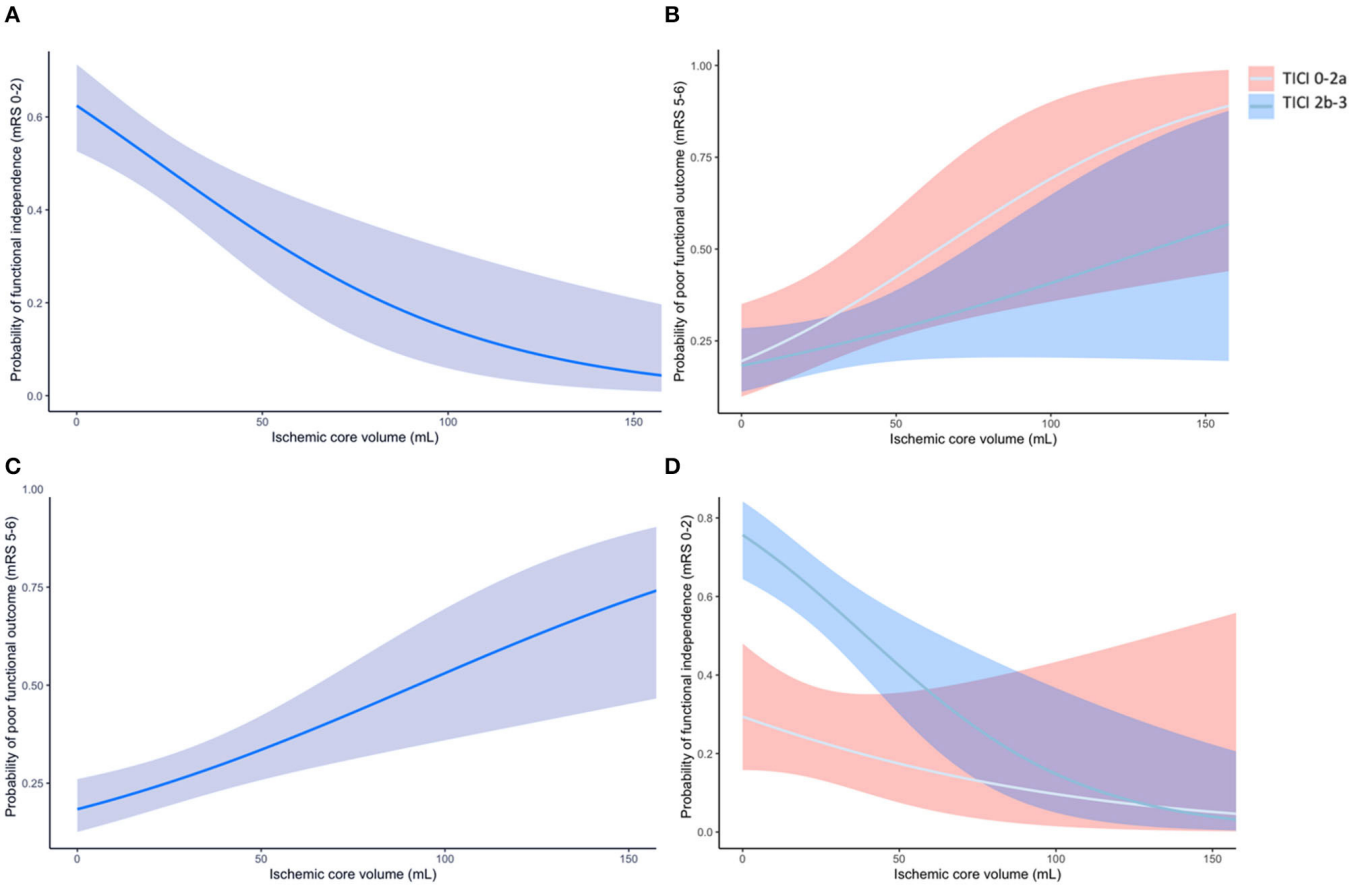
**(A)** The association of CTP ischemic core volume with the probability of poor outcome (mRS 5-6) at 90 days. **(B)** The association of CTP ischemic core volume with the probability of poor outcome for patients with successful reperfusion (eTICI 2b-3; blue line with blue area representing the 95%CI) and unsuccessful reperfusion (eTICI 0-2a; gray line with red area representing the 95%CI). **(C)** The association of CTP ischemic core volume with functional independence (mRS 0-2) at 90 days. **(D)** The association of CTP ischemic core volume with the probability of functional independence for patients with successful and unsuccessful reperfusion. The effect of successful reperfusion (eTICI 2b-3) on achieving functional independence was modified by CTP ischemic core (*p for interaction* < 0.01).

Median CTP ischemic core volume was 13 mL. CTP ischemic core volume was significantly associated with poor outcome in univariable (OR per 10 mL 1.03 [95% CI 1.02–1.05]), and multivariable logistic regression analysis (aOR per 10 mL 1.02 [95% CI 1.01–1.04]) after adjusting for the predefined covariates (Table 2). Larger CTP ischemic core volume was associated with lower likelihood of having a lower mRS at 90 days [aOR per 10 mL 0.85 (95% CI 0.78–0.93)] and a lower chance of achieving functional independence [aOR per 10 mL 0.98 (95%CI 0.96–0.99)]. The association between ischemic core volume as a continuous variable and functional outcome is shown in Figure 2. The effect of successful reperfusion (eTICI2b-3) on ordinal mRS was not modified by CTP ischemic core volume (*P for interaction* 0.84). A detailed presentation of the associations of clinical and imaging and parameters with poor functional outcome is given in Table 2 and Supplementary Table I.

**Table 2:** Univariable and multivariable analysis of imaging parameters with functional outcomes.

	**Poor outcome (mRS 5-6)**				**Lower mRS at 90 days**				**Functional independence (mRS 0–2)**			
	**OR (95%CI)**	***P-*** **value**	**aOR (95%CI)**	***P-*** **value**	**OR (95%CI)**	***P-*** **value**	**aOR (95%CI)**	***P-*** **value**	**OR (95%CI)**	***P-*** **value**	**aOR (95%CI)**	***P-*** **value**
CTP ischemic core volume (per 10 mL)	1.03 (1.02–1.05)	**<0.01**	1.02 (1.01–1.04)	**<0.01**	0.83 (0.78–0.93)	**<0.01**	0.85 (0.78–0.93)	**<0.01**	0.96 (0.94–0.98)	**<0.01**	0.98 (0.96–0.99)	**<0.01**
CTA-CS	0.93 (0.85–1.00)	0.07	0.96 (0.90–1.04)	0.29	1.39 (1.00–1.95)	**0.05**	1.10 (0.75–1.64)	0.54	1.07 (0.97–1.16)	0.13	1.01 (0.91–1.07)	0.90
ASPECTS	0.98 (0.95–1.02)	0.43	0.98 (0.96–1.02)	0.34	1.13 (0.97–1.31)	0.11	1.13 (0.96–1.34)	0.15	1.01 (0.97–1.06)	0.63	1.01 (0.98–1.05)	0.56

*ASPECTS, Alberta Stroke Program Early CT Score; CTA-CS, Computed Tomography Angiography Collateral Score; IVT, intravenous alteplase; mRS, modified Rankin Scale; NIHSS, National Institutes of Health Stroke Scale; (a) OR, (adjusted) Odds Ratio. Bold values represent statistically significant results*.

### Association of CTA-CS With Outcome

CTA-CS was not significantly associated with poor outcome [aOR 0.96 (95% CI 0.90–1.04)] in multivariable analysis (Table 2 and Supplementary Tables I, II). In univariable analysis, CTA-CS was associated with a lower mRS at 90 days [OR 1.39 (95%CI 1.00–1.95)]. This association was not significant after adjusting for the predefined covariates [aOR 1.10 (95%CI 0.75–1.64)]. CTA-CS was not associated with functional independence in univariable or multivariable analyses (Table 2). Poor collaterals were not significantly associated with poor outcome [aOR 1.05 (95%CI 0.91–1.16)]. The effect of successful reperfusion on ordinal mRS at 90 days was not modified by CTA-CS (*P for interaction* 0.22).

### Association of ASPECTS With Outcome

We did not find a statistically significant association between ASPECTS and poor outcome, lower mRS at 90 days, or functional independence in either univariable nor multivariable analysis (Table 2 and Supplementary Tables I, II). Poor ASPECTS (*N* = 13) was not significantly associated with poor outcome [aOR 1.18 (95%CI 0.93–1.46), *p* = 0.15]. The effect of successful reperfusion on ordinal outcome was not modified by ASPECTS (*P for interaction* = 0.36).

### Association of Dichotomized CTP Ischemic Core Volume With Outcome

Exploratory subgroup analysis showed that CTP ischemic core volume ≥70 mL was significantly associated with poor outcome [aOR 1.32 (95%CI 1.14–1.65)]. Twenty-two (11%) of 201 patients had a CTP ischemic core volume ≥70 mL, of which 13 (65%) had poor outcome at 90 days post-EVT, compared to 38 (23%) patients with CTP ischemic core volume <70 mL (*P* < 0.01). Mortality was more common in patients with CTP ischemic core volume ≥70 mL compared to patients with CTP ischemic core volume <70 mL (55 vs. 16%; *P* < 0.01). Two (10%) patients with CTP ischemic core volume ≥70 mL achieved functional independence compared to 88 (49%) patients with CTP ischemic core volume <70 mL (*p* < 0.01; Supplementary Figure III). Symptomatic intracranial hemorrhage occurred more frequently in patients with CTP ischemic core volume ≥70 mL compared to patients with CTP ischemic core volume <70 mL (13 vs. 3%; *p* = 0.01). Results of the subgroup analysis with a threshold of 50 mL are reported in the data supplement (Supplementary Table III, Supplementary Figure IV).

### Target Mismatch Profile

The median penumbra volume was 96 mL and the median mismatch ratio was 6.6. In univariable analyses, penumbra volume was not associated with poor functional outcome [OR 1.00 (95% CI 0.99–1.01)]. On baseline imaging, 175 (87%) patients did have a TMM profile as applied in the EXTEND-IA trial (2). Thirteen (54%) patients without a TMM profile had a poor outcome after 90 days vs. 38 (23%) patients with a TMM profile [OR 1.36 (95% CI 1.13–1.64)]. In the group without a TMM profile, 25% of the patients achieved functional independence compared to 52% in the group with a TMM profile [OR 0.76 (95% CI 0.61–0.94)]. The rate of successful reperfusion was similar in both groups: 71% in the TMM profile group vs. 68% in the group without a TMM profile (*p* = 0.73).

### Association of Imaging Biomarkers With Mortality and SICH

Both CTP ischemic core volume and CTA-CS were significantly associated with mortality and sICH in univariable analyses (Supplementary Table III). In contrast, ASPECTS was not significantly associated with either mortality or sICH.

## Discussion

Our results showed that larger CTP ischemic core volume was associated with poor outcome, a lower likelihood of lower mRS at 90 days, and a lower chance of functional independence in a cohort of patients treated with EVT in daily clinical practice. The relative benefit of successful recanalization – as proxy for EVT – to prevent poor outcome was not modified by CTP ischemic core volume. Our results showed that a better CTA-CS was associated with a higher likelihood of having a lower mRS at 90 days in univariable analysis only. In contrast to CTP ischemic core volume, neither CTA-CS nor ASPECTS were associated with poor outcome, lower mRS at 90 days, or functional independence in multivariable analyses.

Although penumbra volume alone was not associated with outcome independent of ischemic core volume, we found that absence of a favorable perfusion imaging, or target mismatch (TMM) profile, was associated with poor outcome. The subgroup analysis of patients with CTP ischemic core ≥70 or <70 mL showed higher rates of mortality and intracranial hemorrhage in patients with CTP ischemic core volume ≥70 mL. In addition, patients with a CTP ischemic core volume ≥70 mL less often achieved functional independence. This is in line with previous results from the SELECT and TREVO studies (16). Moreover, other studies on the association of CTP ischemic core volume with functional outcome found similar results (17, 18).

Several studies have described the association between CTA-CS and ASPECTS and functional outcome in larger study populations. In a previous study in a larger subset of the MR CLEAN Registry population, good collateral status was found to be strongly associated with better functional outcome of patients with acute ischemic stroke due to intracranial LVO of the anterior circulation (19). Interestingly, this association was not found in our subgroup of patients included in the MR CLEAN Registry who received baseline CTP. The absence of a significant association between CTA-CS and functional outcome in our cohort might be caused by a significantly smaller sample size (201 vs. 1412 patients). Nonetheless, in line with our results, collateral status alone was unable to identify patients who are likely or less likely to benefit from EVT in the study by Jansen et al. (19).

The association between ASPECTS and functional outcome has also been studied in various studies. In a recent *post-hoc* analysis from the MR CLEAN Registry by Ospel et al., a significant association between ASPECTS and functional outcome was found (20). Most probably, the different results between this analysis and our study can be explained by the (much) larger sample size (201 vs. 3279 patients) in their analysis. The benefit of EVT in patients with low ASPECTS has been extensively explored in previous studies (21, 22). In a subgroup analysis of the MR CLEAN trial, ASPECTS 5-7 was associated with functional outcome (relative to ASPECTS 8-10 and ASPECTS 0-4), however no significant modification of treatment effect by ASPECTS was found (21). This is in line with our results, which showed no modification of successful reperfusion. Yet, we should note that only very few (*N* = 13) patients with low ASPECTS scores were included in our analysis.

Although there is no clear consensus yet on the additional value of selecting patients for EVT based on acute stroke imaging biomarkers, such as CTP ischemic core volume, CTA-CS, or ASPECTS, all three imaging biomarkers are commonly used to select patients for EVT and are often incorporated in the exclusion criteria of EVT trials (23, 24). All acute stroke imaging biomarkers have their specific limitations. For example, CTP is sensitive to patient motion, requires appropriate contrast administration, and results differ between processing software packages (15, 25, 26). On the other hand, for both CTA-CS and ASPECTS, the interobserver agreement is a major limiting factor (27, 28).

In our study population, poor outcome in EVT-treated patients was associated with age, NIHSS, and ischemic core volume. Previous studies have extensively developed prognostic scores or models to predict poor outcome prior to reperfusion therapy (13, 29). In the current transition toward more personalized acute stroke care, a clinically validated prediction model, such as MR PREDICTS, could support physicians in clinical decision-making based on patient-based physiological characteristics (29). Most current models, however, do not take CTP characteristics into account. Our results suggest that CTP ischemic core volume might be a valuable addition to such prognostic models to further improve their predictive power.

Several limitations to our study should be noted. First, only data of patients who underwent EVT were recorded in the MR CLEAN Registry. Therefore, we were not able to compare our results with a formal control group that did not undergo EVT. Instead, we used unsuccessful reperfusion as a proxy for the control group in our analysis. Second, the MR CLEAN Registry had no prespecified diagnostic imaging criteria for patient selection, therefore it is possible that patients presenting with poor clinical and imaging profiles were not considered as EVT candidates. Also, selection bias might have been introduced since no standardized CTP acquisition was required for inclusion in the MR CLEAN Registry (19% had baseline CTP) and was therefore not always performed according to the same protocol in all participating centers – if performed routinely at all. However, a comparison of the study population with the overall MR CLEAN Registry cohort showed no differences in the baseline distribution of ASPECTS or CTA-CS between the two groups. Third, CTA-CS was scored on single-phase CTA images. Therefore, our results might not be applicable to a setting in which multiphase CTA images are acquired. Finally, in this study, we assessed the association of commonly used acute stroke imaging biomarkers separately. Therefore, our results cannot be compared with performances of prognostic models using a combination of acute stroke imaging biomarkers. Future studies could potentially explore the performance of a prognostic model using a combination of acute stroke imaging biomarkers to further improve the outcome prediction in acute ischemic stroke patients.

It has been suggested that patients with fast core growth—and thus a higher chance of a larger core volume—are more likely to present early (30). Yet, this was not reflected by our study population as our patient sample—along with the overall MR CLEAN Registry cohort—included only a few patients with ischemic core volumes ≥70 mL. A possible explanation could be that patients with an estimated ischemic core volume ≥70 mL were excluded from treatment, although we could not confirm this. To date, the efficacy of EVT in patients with larger ischemic core volumes remains a topic of debate. Although a potential benefit from EVT is suggested in patients with larger core volumes, evidence is mainly based on non-randomized data (31). Most patients were enrolled before publication of the DAWN and DEFUSE-3 results. Therefore, our cohort consisted mainly of patients who were treated in the 0–6 h time window, which might underestimate the discriminating power of CTP in patient selection, especially in the 6–24 h window. Finally, our results might not be applicable to imaging data that are post-processed using different software. Previous studies have shown that different CTP post-processing software packages give different results, although these differences were less severe in patients with CTP ischemic core volumes <70 mL (15, 26).

## Conclusion

In anterior circulation acute ischemic stroke patients treated with EVT in daily clinical practice, larger CTP ischemic core volume is associated with poor outcome, a lower likelihood of having a lower mRS at 90 days, and a lower probability of functional independence. ASPECTS and CTA-CS are not significantly associated with poor outcome or the chance of having a lower mRS at 90 days. None of the imaging biomarkers provided a strong argument to withhold EVT.

## Data Availability Statement

The datasets presented in this article are not readily available since individual patient data cannot be made available under Dutch law if no consent was obtained.

## Ethics Statement

The studies involving human participants were reviewed and approved by Central Medical Ethics Committee of the Erasmus MC, Rotterdam, Netherlands. The Ethics Committee waived the requirement of written informed consent for participation.

## Author Contributions

MSK and JH collected and prepared data for this study, performed the statistical analysis, interpreted the results, and drafted the paper. HM, BE, OB, and CM assisted with the statistical analysis and interpretation of the results. MK, OB, LB, WZ, HJ, JD, DD, JC, HM, BE, and CM critically revised the paper. All authors contributed to the article and approved the submitted version.

## Funding

The MR CLEAN Registry was funded and carried out by the Erasmus University Medical Center, the Academic Medical Center Amsterdam, and the Maastricht University Medical Center. The MR CLEAN Registry was additionally funded by the Applied Scientific Institute for Neuromodulation (TWIN).

## Conflict of Interest

BE reports grants from LtC (ZonMW and TKI-PPP of Health Holland outside the submitted work. WZ reports personal fees from Cerenovus and from Stryker outside the submitted work. DD reports grants from the Dutch Heart Foundation, AngioCare, Medtronic/Covidien/EV3, MEDAC/LAMEPRO, Penumbra, Top Medical/Concentric, and Stryker during conduct of the study; consultation fees from Stryker, Bracco Imaging, and Servier, received by the Erasmus University Medical Centre, outside the submitted work. CM reports grants from TWIN, during the conduct of the study and grants from CVON/Dutch Heart Foundation, European Commission, Dutch Health Evaluation Program, and from Stryker outside the submitted work (paid to institution) and is shareholder of NICO.LAB. HM is co-founder and shareholder of NICO.LAB. The remaining authors declare that the research was conducted in the absence of any commercial or financial relationships that could be construed as a potential conflict of interest.

## Publisher's Note

All claims expressed in this article are solely those of the authors and do not necessarily represent those of their affiliated organizations, or those of the publisher, the editors and the reviewers. Any product that may be evaluated in this article, or claim that may be made by its manufacturer, is not guaranteed or endorsed by the publisher.

## Abbreviations

ASPECTS: Alberta Stroke Program Early CT Score
CTA: Computed Tomography Angiography
CTA-CS: Computed Tomography Angiography Collateral Score
CTP: Computed Tomography Perfusion
IQR: Interquartile Range
EVT: Endovascular Thrombectomy
LVO: Large Vessel Occlusion
MR CLEAN: Multicenter Randomized Clinical Trial of Endovascular Treatment for Acute Ischemic Stroke in the Netherlands
mRS: modified Rankin Scale
OR: Odds Ratio
TICI: Thrombolysis in Cerebral Infarction.
