# Supplementary material for: Association of Ischemic Core Imaging Biomarkers With Post-Thrombectomy Clinical Outcomes in the MR CLEAN Registry
Source: Front Neurol. 2022 Jan 10;12:771367. doi: 10.3389/fneur.2021.771367 (PMC8784730; doi:10.3389/fneur.2021.771367)
Supplement: Supplementary file 1 [file Data_Sheet_1.pdf]

SUPPLEMENTAL MATERIAL

Appendix A

Supplemental Figure I. Distribution of CTP ischemic core volume (CBV <1.2 mL/100mL) for each Alberta Stroke Program Early CT Score (ASPECTS) category. The estimated core volume on perfusion imaging varied within the same ASPECTS.

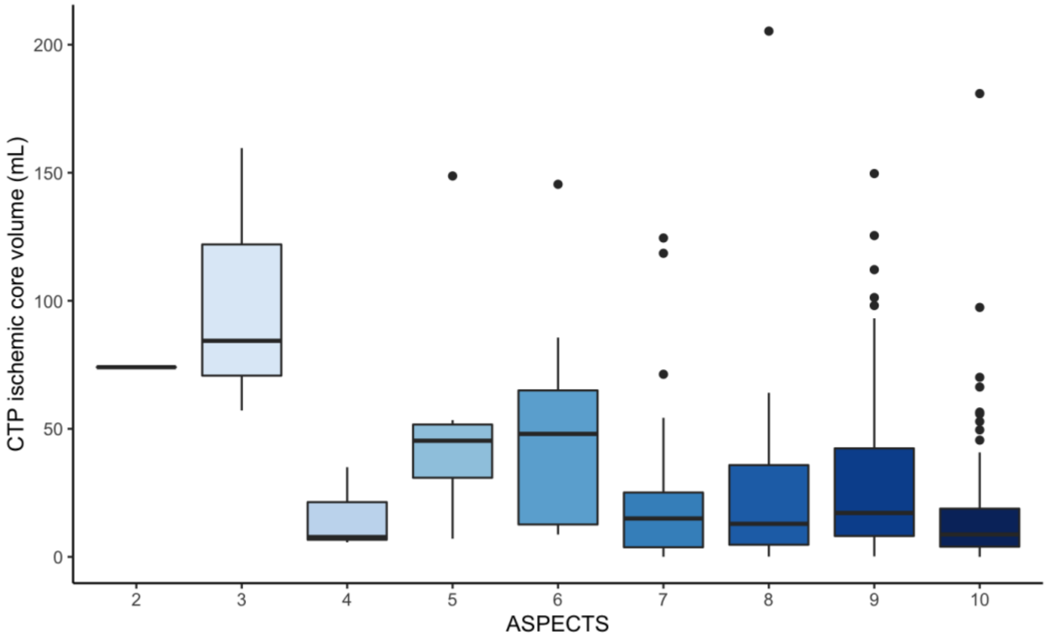

Supplemental Figure II. Distribution of CTP ischemic core volume (CBV <1.2 mL/100mL) for each CTA collateral score (CTA-CS) category. CTP ischemic core volumes were larger in patients with lower collateral scores. A higher degree of discordance was observed for lower CTA-CS with larger CTP ischemic core volumes.

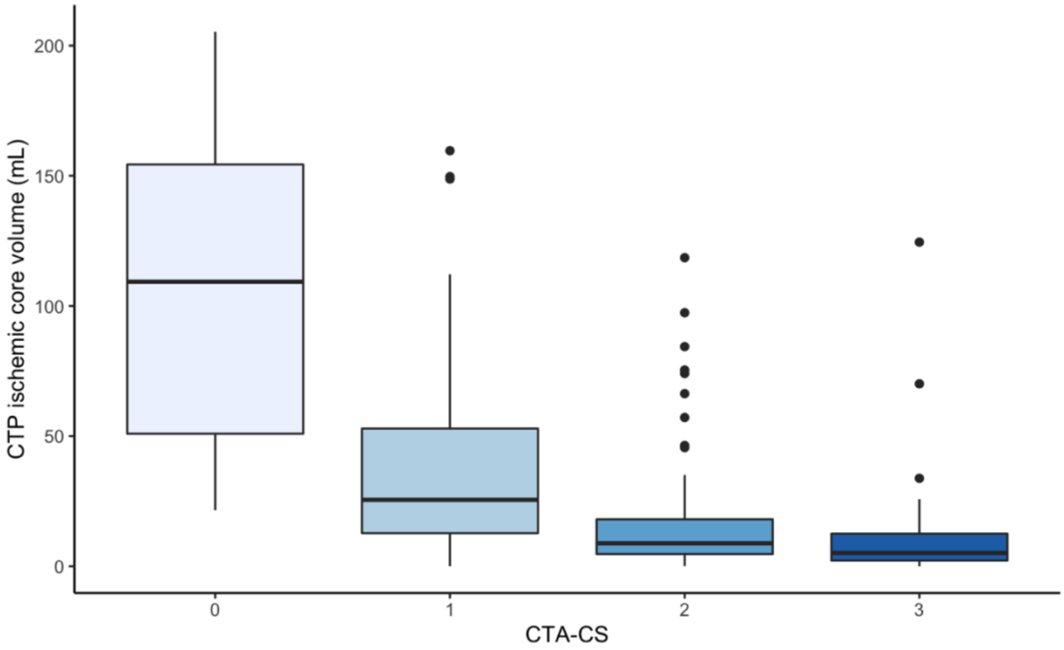

**Supplemental Table I. Univariable analysis of poor functional outcome (mRS 5-6).** ASPECTS, Alberta Stroke Program Early CT Score; CTA-CS; Computed Tomography Angiography Collateral Score; IVT, intravenous alteplase; mRS, modified Rankin Scale; NIHSS, National Institutes of Health Stroke Scale; OR, Odds Ratio.		
**Variables**	**OR (95% CI)**	***P* value**
**CTP ischemic core volume (per 10 mL)**	**1.03 (1.02-1.05)**	**<0.01**
**CTA-CS**	**0.93 (0.85-1.00)**	**0.07**
**ASPECTS**	**0.98 (0.95-1.02)**	**0.43**
Age (per year)	0.93 (0.85-1.00)	0.07
NIHSS (per point)	0.98 (0.95-1.02)	0.43
Male sex (vs. female)	0.88 (0.78-1.00)	0.04
IVT	0.98 (0.85-1.12)	0.70
Onset-to-groin time (per 10 min)	1.01 (1.00- 1.01)	0.06
Pre-mRS	1.16 (1.10-1.21)	<0.01
Occlusion location	0.94 (0.86-1.02)	0.15

Supplemental Table II. Multivariable analysis of poor functional outcome (mRS 5-6). ASPECTS, Alberta Stroke Program Early CT Score; CTA-CS; Computed Tomography Angiography Collateral Score; IVT, intravenous alteplase; mRS, modified Rankin Scale; NIHSS, National Institutes of Health Stroke Scale; aOR, adjusted Odds Ratio.

**Variables**		**aOR (95% CI)**	***P* value**
**CTP ischemic core volume (per 10 mL)**		**1.03(1.02-1.05)**	**<0.01**
	NIHSS (per point)	1.00 (0.99-1.01)	0.93
	Pre-mRS	1.11 (1.05-1.16)	< 0.01
	Male sex (vs. female)	0.90 (0.81-1.00)	0.09
	Age (per year)	1.01 (1.01-1.01)	<0.01
	IVT	1.07 (0.94-1.20)	0.23
	Onset-to-groin time (per 10 min)	1.00 (0.99-1.01)	0.08
	Occlusion location	0.96 (0.88-1.02)	0.34
**CTA-CS**		**0.96 (0.90-1.04)**	**0.29**
	NIHSS (per point)	1.00 (0.99-1.01)	0.56
	Pre-mRS	1.12 (1.06-1.17)	<0.01
	Male sex (vs. female)	0.93 (0.83-1.03)	0.20
	Age (per year)	1.01 (1.01-1.01)	<0.01
	IVT	1.08 (0.95-1.21)	0.22
	Onset-to-groin time (per 10 min)	1.00 (1.00-1.01)	0.10
	Occlusion location	0.95 (0.87-1.01)	0.19
**ASPECTS**		**0.98 (0.96-1.02)**	**0.34**
	NIHSS (per point)	1.00 (0.99-1.01)	0.40
	Pre-mRS	1.11 (1.06-1.16)	<0.01
	Male sex (vs. female)	0.93 (0.83-1.03)	0.17
	Age (per year)	1.01 (1.01-1.01)	<0.01
	IVT	1.09 (0.96-1.22)	0.16
	Onset-to-groin time (per 10 min)	1.00 (1.00-1.01)	0.16
	Occlusion location	0.95 (0.87-1.01)	0.21

Supplemental Figure III. Distribution of the modified Rankin Scale (mRS) scores at 90 days for patients with CTP ischemic core volumes ≥70 mL versus <70mL.

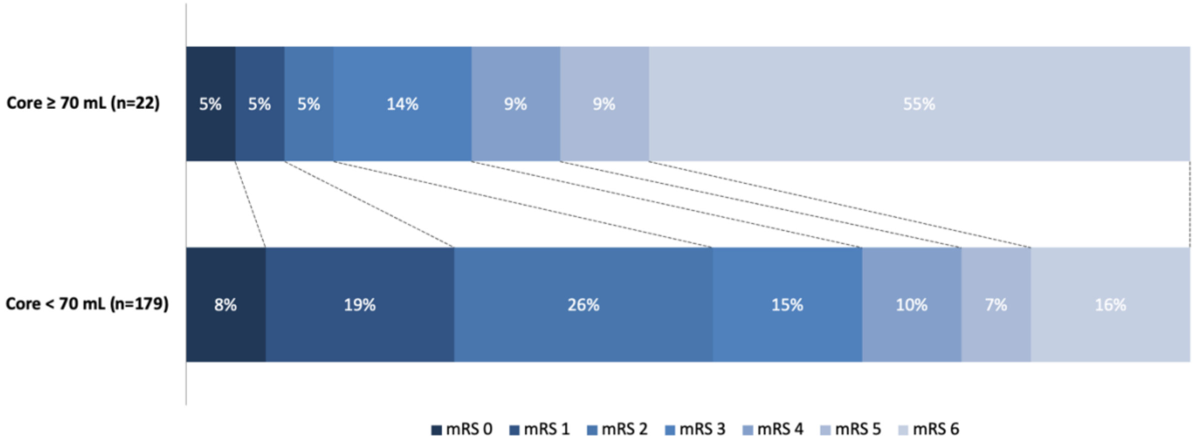

Supplemental Figure IV. Distribution of the modified Rankin Scale (mRS) scores at 90 days for patients with CTP ischemic core volumes ≥50 mL versus <50mL.

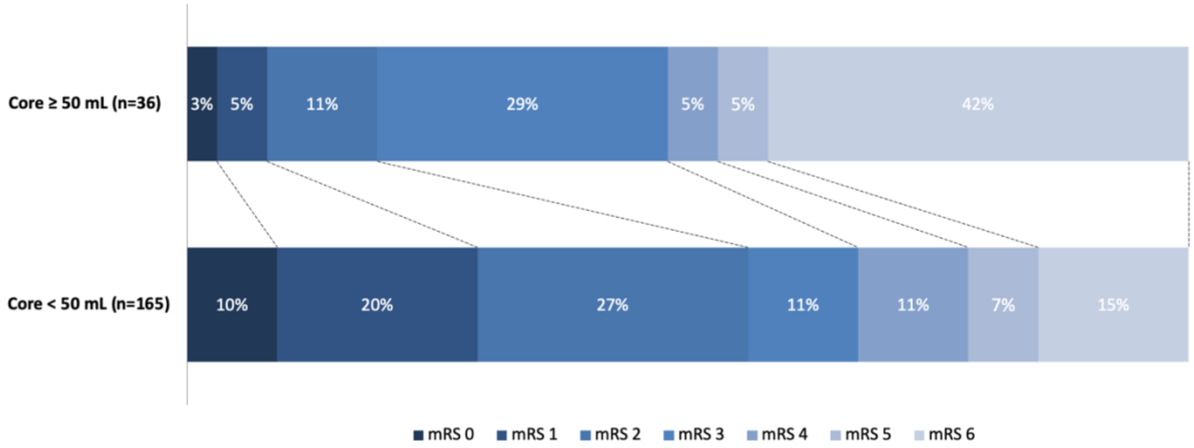

Supplemental Table III. Functional outcome (mRS) at 90 days stratified by ischemic core volume (≥50 mL versus <50mL). mRS, modified Rankin Scale

	**≥50 mL**	**<50mL**	***P* value**
Poor outcome (mRS 5-6) – n(%)	17(50)	34(22)	**<0.01**
Functional independence (mRS 0-2) – n(%)	4(12)	86(56)	**<0.01**
Mortality– n(%)	15(44)	23(15)	**<0.01**
sICH– n(%)	4(11)	4(2)	0.02

Supplemental Table IV. Association of imaging biomarkers with mortality and sICH. ASPECTS,Alberta Stroke Program Early CT Score; mRS,modified Rankin Scale; aOR,adjusted Odds Ratio.

	**Mortality**		**sICH**	
	aOR(95%CI)	*P* value	aOR(95%CI)	*P* value
CTP ischemic core volume (per 10 mL)	1.03(1.01-1.04)	**<0.01**	1.01(1.01-1.02)	**<0.01**
CTA-CS	0.95(0.88-1.02)	0.14	0.96(0.92-0.99)	**0.02**
ASPECTS	0.98(0.95-1.01)	0.22	0.99(0.97-1.01)	0.15

Appendix B

MR CLEAN Registry Investigators – group authors

Executive committee

Diederik W.J. Dippel[1];Aad van der Lugt[2];Charles B.L.M. Majoie[3];Yvo B.W.E.M. Roos[4];Robert J. van Oostenbrugge[5];Wim H. van Zwam[6];Jelis Boiten[14];Jan Albert Vos[8]

Study coordinators

Ivo G.H. Jansen[3];Maxim J.H.L. Mulder[1,2];Robert- Jan B. Goldhoorn[5,6];Kars C.J. Compagne[2];Manon Kappelhof[3];Josje Brouwer[4];Sanne J. den Hartog[1,2,40];Wouter H. Hinsenveld [5,6];

Local principal investigators

Diederik W.J. Dippel[1];Bob Roozenbeek[1];Aad van der Lugt[2];Adriaan C.G.M. van Es[2];Charles B.L.M. Majoie[3];Yvo B.W.E.M. Roos[4];Bart J. Emmer[3];Jonathan M. Coutinho[4];Wouter J. Schonewille[7];Jan Albert Vos[8]; Marieke J.H. Wermer[9];Marianne A.A. van Walderveen[10];Julie Staals[5];Robert J. van Oostenbrugge[5];Wim H. van Zwam[6];Jeannette Hofmeijer[11];Jasper M. Martens[12];Geert J. Lycklama à Nijeholt[13];Jelis Boiten[14];Sebastiaan F. de Bruijn[15];Lukas C. van Dijk[16];H. Bart van der Worp[17];Rob H. Lo[18];Ewoud J. van Dijk[19];Hieronymus D. Boogaarts[20];J. de Vries[22];Paul L.M. de Kort[21]; Julia van Tuijl[21] ; Jo P. Peluso[26];Puck Fransen[22];Jan S.P. van den Berg[22];Boudewijn A.A.M. van Hasselt[23];Leo A.M. Aerden[24];René J. Dallinga[25];Maarten Uyttenboogaart[28];Omid Eschgi[29];Reinoud P.H. Bokkers[29];Tobien H.C.M.L. Schreuder[30];Roel J.J. Heijboer[31];Koos Keizer[32];Lonneke S.F. Yo[33];Heleen M. den Hertog[22];Tomas Bulut[35]; Paul J.A.M. Brouwers[34]

Imaging assessment committee

Charles B.L.M. Majoie[3](chair);Wim H. van Zwam[6];Aad van der Lugt[2];Geert J. Lycklama à Nijeholt[13];Marianne A.A. van Walderveen[10];Marieke E.S. Sprengers[3];Sjoerd F.M. Jenniskens[27];René van den Berg[3];Albert J. Yoo[38];Ludo F.M. Beenen[3];Alida A. Postma[6];Stefan D. Roosendaal[3];Bas F.W. van der Kallen[13];Ido R. van den Wijngaard[13];Adriaan C.G.M. van Es[2];Bart J. Emmer[,3];Jasper M. Martens[12]; Lonneke S.F. Yo[33];Jan Albert Vos[8]; Joost Bot[36], Pieter-Jan van Doormaal[2]; Anton Meijer[27];Elyas Ghariq[13]; Reinoud P.H. Bokkers[29];Marc P. van Proosdij[37];G. Menno Krietemeijer[33];Jo P. Peluso[26];Hieronymus D. Boogaarts[20];Rob Lo[18];Dick Gerrits[35];Wouter Dinkelaar[2]Auke P.A. Appelman[29];Bas Hammer[16];Sjoert Pegge[27];Anouk van der Hoorn[29];Saman Vinke[20].

Writing committee

Diederik W.J. Dippel[1](chair);Aad van der Lugt[2];Charles B.L.M. Majoie[3];Yvo B.W.E.M. Roos[4];Robert J. van Oostenbrugge[5];Wim H. van Zwam[6];Geert J. Lycklama à Nijeholt[13];Jelis Boiten[14];Jan Albert Vos[8];Wouter J. Schonewille[7];Jeannette Hofmeijer[11];Jasper M. Martens[12];H. Bart van der Worp[17];Rob H. Lo[18]

Adverse event committee

Robert J. van Oostenbrugge[5](chair);Jeannette Hofmeijer[11];H. Zwenneke Flach[23]

Trial methodologist

Hester F. Lingsma[40]

Research nurses / local trial coordinators

Naziha el Ghannouti[1];Martin Sterrenberg[1];Wilma Pellikaan[7];Rita Sprengers[4];Marjan Elfrink[11];Michelle Simons[11];Marjolein Vossers[12];Joke de Meris[14];Tamara Vermeulen[14];Annet Geerlings[19];Gina van Vemde[22];Tiny Simons[30];Gert Messchendorp[28];Nynke Nicolaij[28];Hester Bongenaar[32];Karin Bodde[24];Sandra Kleijn[34];Jasmijn Lodico[34]; Hanneke Droste[34];Maureen Wollaert[5];Sabrina Verheesen[5];D. Jeurrissen[5];Erna Bos[9];Yvonne Drabbe[15];Michelle Sandiman[15];Nicoline Aaldering[11];Berber Zweedijk[17];Jocova Vervoort[21];Eva Ponjee[22];Sharon Romviel[19];Karin Kanselaar[19];Denn Barning[10].

PhD / Medical students:

Esmee Venema[40]; Vicky Chalos[1,40]; Ralph R. Geuskens[3]; Tim van Straaten[19];Saliha Ergezen[1]; Roger R.M. Harmsma[1]; Daan Muijres[1]; Anouk de Jong[1];Olvert A. Berkhemer[1,3,6];Anna M.M. Boers[3,39]; J. Huguet[3];P.F.C. Groot[3];Marieke A. Mens[3];Katinka R. van Kranendonk[3];Kilian M. Treurniet[3];Manon L. Tolhuisen[3,39];Heitor Alves[3];Annick J. Weterings[3],Eleonora L.F. Kirkels[3],Eva J.H.F. Voogd[11];Lieve M. Schupp[3];Sabine L. Collette[28,29];Adrien E.D. Groot[4];Natalie E. LeCouffe[4];Praneeta R. Konduri[39];Haryadi Prasetya[39];Nerea Arrarte-Terreros[39];Lucas A. Ramos[39].

List of affiliations

Department of Neurology[1], Radiology[2], Public Health[40], Erasmus MC University Medical Center;
Department of Radiology and Nuclear Medicine[3], Neurology[4], Biomedical Engineering & Physics[39], Amsterdam UMC, University of Amsterdam, Amsterdam;
Department of Neurology[5], Radiology[6], Maastricht University Medical Center and Cardiovascular Research Institute Maastricht (CARIM);
Department of Neurology[7], Radiology[8], Sint Antonius Hospital, Nieuwegein;
Department of Neurology[9], Radiology[10], Leiden University Medical Center;
Department of Neurology[11], Radiology[12], Rijnstate Hospital, Arnhem;
Department of Radiology[13], Neurology[14], Haaglanden MC, the Hague;
Department of Neurology[15], Radiology[16], HAGA Hospital, the Hague;
Department of Neurology[17], Radiology[18], University Medical Center Utrecht;
Department of Neurology[19], Neurosurgery[20], Radiology[27], Radboud University Medical Center, Nijmegen;
Department of Neurology[21], Radiology[26], Elisabeth-TweeSteden ziekenhuis, Tilburg;
Department of Neurology[22], Radiology[23], Isala Klinieken, Zwolle;
Department of Neurology[24], Radiology[25], Reinier de Graaf Gasthuis, Delft;
Department of Neurology[28], Radiology[29], University Medical Center Groningen;
Department of Neurology[30], Radiology[31], Atrium Medical Center, Heerlen;
Department of Neurology[32], Radiology[33], Catharina Hospital, Eindhoven;
Department of Neurology[34], Radiology[35], Medisch Spectrum Twente, Enschede;
Department of Radiology[36], Amsterdam UMC, Vrije Universiteit van Amsterdam, Amsterdam;
Department of Radiology[37], Noordwest Ziekenhuisgroep, Alkmaar;
Department of Radiology[38], Texas Stroke Institute, Texas, United States of America.
